# On the Reflex Function of the Brain

**Published:** 1845-01

**Authors:** T. Laycock

**Affiliations:** Physician to the York Dispensary, &c.


### ON THE REFLEX FUNCTION OF THE BRAIN.

BY T. LAYCOCK, M.D.,
Physician to the York Dispensary, &c.

[*Read at York, before the Medical Section of the British Association for the Advancement of Science, on 28th Sept.* 1844.]

SINCE it has been generally acknowledged that the brain is the organ of mind, the study of its physiology or laws of action, has acquired a surpassing interest, for whatever men do, in the most comprehensive sense, is connected with its functions. It is, however, as elucidating the nature and treatment of insanity, that its physiology is most interesting to the physician.

A knowledge of the laws and mode of action of this important organ can only be acquired by scientific observation and induction, and it is encouraging and pleasing to know that the multitude and variety of facts from which inductions may be made are proportionate to the difficulties to be overcome. I am not alluding to mental philosophy, but to the advances already accomplished in comparative physiology, which show us that the structure and functions of the nervous system in all animals are subject to the same laws of development and action; that a continuous and harmonious whole is formed out of the multitudinous and disjected parts; and that varied and dissimilar as they appear, each may be made to illustrate the other.

Four years have elapsed since I published my opinion, supported by such arguments as I could then state, that the brain, although the organ of consciousness, was subject to the laws of reflex action, and that in this respect it did not differ from the other ganglia of the nervous system. I was led to this opinion by the general principle, that the ganglia within the cranium being a continuation of the spinal cord, must necessarily be regulated as to their reaction on external agencies by laws identical with those governing the functions of the spinal ganglia and their analogues in the lower animals. And I was confirmed in this opinion by finding, after the investigation and collocation of known facts, that observations and arguments like those satisfactorily adduced in proof of the existence of the reflex function of the spinal ganglia, may be brought forward in proof that the cerebral ganglia have similar endowments. In the present paper I purpose to give these proofs connectedly. I must premise, however, that I entered upon my undertaking with considerable hesitation. I felt deeply the magnitude of the subject, and the important results to which an inquiry of this kind might lead. I felt too, that in advocating the doctrine of cerebral reflex function, I was opposing the opinions of a physiologist to whom deference is eminently due. That gentleman, however, is so devoted to all questions of neurology, and so anxious, I really believe, to arrive at truth, that he, I hope, will willingly permit me to differ from him in doctrine, and give a favorable consideration to my opinions, although opposed to his own.

To render my subsequent arguments clearer, I will first give a short summary of the doctrine of reflex action, as at present received. I need scarcely state that the spinal cord in the vertebrata, is a series of ganglia analogous to those of the articulata. If a centipede be divided into several parts, each segment will move on an external stimulus being applied; if it be decapitated, and the respiratory orifices on one side of the body be irritated by an acrid vapour, it will immediately flex the trunk to the opposite side; if the Geophilus electricus be cut into two pieces, each segment will live and appear vigorous for a fortnight, the caudal portion surviving the cephalic for two or three days. Cold-blooded vertebrates display these involuntary motions very strikingly; thousands of unfortunate frogs have fallen victims to the zeal of physiologists in researches of this kind. If the brain, the organ of consciousness, be removed from a frog by decapitation, it will still attempt to escape when pinched or otherwise injured, and will perform motions, which, if the brain had not been removed, could only have been supposed to be the result of sensation and volition. Indeed it has been inferred from these facts, that the spinal cord, as well as the brain, is endowed with consciousness. It is found, however, that *segments* of the spinal cord possess a similar function. If that portion of the spinal axis of a frog which gives origin to the brachial nerves be separated both anteriorly and posteriorly from the whole cord, so as to be completely isolated, on stimulating the skin covering the forelegs, retraction of the limb irritated and similar reflex movements take place. There can be no doubt, in fact, that each ganglion constitutes the centre of a nervous arc, of which the motor and sensitive nerves, in connexion with it are the two limbs. An impression is made on the peripheral termination of a sensitive nerve; this impression is transmitted to the ganglion as the central axis; there some change, the nature of which is unknown, takes place, but such, that the muscles in connexion with the motor nerves arising from the ganglion are moved. Dr. Hall has termed the sensitive, or afferent, or impression-bearing nerve, incident excitor nerve; and the central axes form collectively the true spinal system, extending from the corpora quadrigemina to the cauda equina.

It is not necessary to reflex action that the irritation be applied exclusively to the peripheral termination of the sensitive or incident excitor nerve, although phenomena so induced, are the most strikingly and most perfectly reflex. The irritation may be applied in any portion of its course, or to the posterior gray matter of the cord (the sensory track) in which the nerve terminates, or to the anterior motor track, or to the cut end of that portion of the motor nerve still in connexion with the muscle. The track of the irritation is from the surface to the muscular fibre through the ganglia; if the continuity of nervous connexion be broken, the irritation cannot reach the muscle.

Irritations may in their origin, therefore, be either peripheral, or derived from the surface; fibrillar, or seated in the trunk of the nerve; and central, or in the axis itself. These variations as to the point from which the irritation commences are connected with variations in the phenomena, and enabled Dr. Hall to arrange excito-motory actions into classes. In hydrophobia, the irritation is a poison circulating with the blood through the central axis; the disease is therefore termed centric, and the excito-motory phenomena are of centric origin; so also the convulsions (the excito-motory phenomena) of asphyxia are centric, because they depend on the circulation of venous blood, instead of arterial, through the spinal cord. The action of the respiratory muscles in sneezing induced by irritation of the schneiderian membrane is reflex, and so with a multitude of vital acts, all admirably elucidated by the law of reflex action.

Motor phenomena, when purely reflex, are of course altogether independent of sensation, or perception, or volition, or consciousness. The mind has no part whatever in their causation or course. A person may, however, be conscious of the reflex acts, and unconscious of the irritation which causes them, as in vomiting from renal irritation, or when the legs of paraplegic patients are jerked on irritating the soles; sensation may also accompany reflex phenomena and volition modify them. Thus, ipecacuan acting on the incident excitor nerves of the stomach will produce the reflex act of vomiting accompanied with the sensation of nausea; or tartar-emetic, circulating with the blood in the medulla oblongata, will induce like phenomena, differing only in being of centric origin, while the others are peripheral. If the soles of the feet be tickled, the legs jerk involuntarily and spasmodically by reflex action, but the sensation of tickling is also perceived. In these and similar instances, volition is often unable to restrain or even to modify the reflex acts. The resulting movements are strictly involuntary. I would particularly call attention to this fact as of some importance in understanding the nature of those reflex acts which I shall adduce as being of cerebral origin, for if any movements of this kind be shown to be strictly involuntary, they must necessarily be considered as reflex excited acts accompanied by sensation—acts which the patient is not merely mentally unwilling, but physically unable, to restrain or modify.

Another remarkable characteristic of reflex phenomena is the harmony of movement in the muscles excited into action by irritation traversing the incident excitor nerves, especially from the periphery. The object of all the purely reflex physiological acts is the conservation of the individual, or of the race. In the words of Dr. Hall, every physiological act of the reflex excito-motor power is obviously designed. If the mucous membrane or skin be irritated, the muscles combine to remove the irritation. This is seen in the two different acts of sneezing and coughing, and also in vomiting. When the tail of a decapitated tortoise is irritated, the hind feet are protruded towards the part, with the object apparently of removing the irritation; if the caudal portion of a bisected scolopendra be irritated, it is immediately erected, and the usual threatening position of the creature when irritated, is assumed. From numerous experiments, especially those of Van Deen and Stilling, it is certain, not only that these acts are independent of the will, but dependent on a special arrangement of the constituent fibrils of the spinal cord. According to Stilling, the posterior gray matter is the portion of the spinal ganglia, on and through which the incident excitor impressions impinge and are diffused, the anterior gray matter, the part in which the necessary arrangements for the harmonious action of the muscles are perfected. These facts are of importance to be remembered in defining the cerebral reflex phenomena, and indeed in detecting the modus operandi of the encephalon as the organ of mind. I have already shown how certain strictly involuntary movements are partly reflex: here we learn how certain strictly instinctive acts may be purely reflex, even when coincident with consciousness.

Another circumstance connected with the purely reflex acts, is that their continued performance is unaccompanied by fatigue. I do not attach much importance to this circumstance, except as proving that volition is closely connected with muscular sensation, but it will have its use in elucidating reflex cerebral phenomena. This incapacity of fatigue is shown in the respiratory muscles; and in the long migratory flights which the instinct of birds compels them to take, as ingeniously suggested by Dr. Hall.

Having thus sketched the history of reflex action, I have now to prove by a series of facts similar to those already stated, that the brain and cerebral nerves are also subject to its laws. I have to show that the cerebral nerves, but especially the optic, acoustic, and olfactory, are incident excitor nerves; that impressions made on them will pass on to the central axis, and there induce the necessary changes in the posterior gray matter, or what is analogous thereto in the cerebrum, and thence impinge on the motor-nerves, giving rise to combined muscular acts, or irregular and spasmodic movements. I have to show that similar acts may have a centric origin, that is, that the exciting cause may be *within* the brain, just as the cause of acknowledged reflex acts may be *within* the true spinal cord. I have also to show that these acts are instinctive in their nature.

Every nerve has its peculiar endowments, and its own machinery of action within the central axis. This is true even of those of the surface—the "true spinal" nerves,—which carry the sensations of heat and cold, and of pain from pricking, tearing, or other mechanical stimuli, for all reflex acts are more decided when the tactile apparatus is irritated. It has been comparatively easy to experiment on these, because their ordinary excitants are readily applied to them; but the optic, olfactory, and acoustic nerves are utterly insensible to stimuli of this kind. Pricking or tearing them, or burning them with strong acids would in no degree excite changes like those induced in the retina by light, after traversing an exquisitely constructed optical instrument; nor excite changes in the acoustic nerve, like those produced by the undulatory strokes of the atmosphere, curiously modified in the auditory apparatus. Experiments on the termination or trunk of these nerves, similar to those made on the nerves of the general surface would therefore be useless. The nearest approach is where galvanic action excites flashes of light, or an acid taste. Sounds duly modified must impinge on the auditory nerve, light duly modified on the optic nerve, if we would ascertain their excitor powers; and physiology and pathology can only supply suitable facts. The symptoms of hydrophobia will perhaps best present the required illustration. In hydrophobia, as in poisoning by strychnia, a poison acting on the blood performs the office of a physiological microscope. But there is this difference, that the former exalts the functions of the sensory track, the operation of strychnia is confined to the motor. The symptoms of this disease constitute a series of excited motor acts, observed with sufficient accuracy, and so well marked as to leave no doubt of their character. The excito-motor nerves whose functions are disordered, are (according to Dr. Hall's views) the trifacial and glosso-pharyngeal, the pharyngeal and laryngeal branches of the pneumogastric, and in some instances the posterior spinal nerves. The reflex motor are the motor branches of the fifth, and of the pneumogastric, and the spinal accessory, and other spinal respiratory nerves. The phenomena excited are spasm of the respiratory muscles, and gasping, convulsions of the face, and occasionally of the trunk or limbs, and an extraordinary development of the instinct of conservation. The patient is ever on the watch, and distrusting all around him.

The true state of the lungs in the hydrophobic gasp appears to be that of complete vacuity of air; and hence the distressing sensation of *want of breath*, or the *besoin de respirer*. That this is the fact appears from a consideration of the phenomena themselves; but in a case related by Dr. Babington, (Vide ‘Records and Researches of a private Medical Association,’ p 117; London, 1798,) the patient having been put into a warm-bath had a convulsive gasp just when being taken out, and immediately sank to the bottom, and as Dr. Babington states, would have been "suffocated" or drowned, if immediate assistance had not been given; thus proving that the lungs at the moment of the convulsion were emptied of air. Now to do this, the contractile tissues of the lungs themselves must be brought into energetic action, as well as the muscles of respiration, so that the excito-motory phenomena of hydrophobia extend to the muscular fibres of the air-passages.

The acknowledged excito-motory phenomena of hydrophobia may be induced, firstly, through the sensual nerves of touch, as by the contact of water with the surface of the head, hands, chest, the lips and pharynx; 2d, by a current of air impinging on the face or chest. In the majority of cases, the slightest breath of air will bring on gasping and convulsions. These causes act undoubtedly on the incident nerves mentioned. But, thirdly, a bright surface, as a mirror; fourthly, the sight of water; or fifthly, the sound of water dropping; or sixthly, the idea of water, as when it is suggested to the patient that he shall drink; all most indubitably induce excito-motory phenomena as decided and distinct as the first and second causes. Here we have three classes of irritations inducing the reflex acts of gasping and spasm of the respiratory muscles: 1, the contact of water and air with the surface of the face, chest and mouth; 2, the contact of reflected light with the retina; 3, an idea excited by the sound of water dropping, or by the mention of water. I need scarcely remark that the dreadfully painful gasp in hydrophobia is strictly involuntary. The following examples may be mentioned as illustrative of these statements.

*Effects of the contact of reflected light with the retina.* "On Monday, the 26th of September, at half-past nine in the morning, a looking-glass being presented, to her she jumped off her mother's knee in great agitation, and became convulsed." (Case of Eliza Kittle, aged 3 years, by Sir A. Carlisle, in Gilman's ‘Prize Essay on the Bite of a Rabid Animal,’ p. 171.)—"When a mirror was presented to him he complained in a few seconds of its hurting his eyes. The same convulsive sobbing took place as in the attempt to swallow water, and he turned his head aside with great expression of fear. I gave him money to induce him to look at it a second time, and endeavoured to gain his attention by desiring him to point out to me by the mirror, which of the sores had given him the greatest uneasiness at the time of dressing them; but before he had looked in it a minute, the same effect was produced as before." (Case of John Dyke, aged 9 years, by Dr. Beddoes, 'Med. and Phys. Jour. vol. xx, p. 196.)

*The idea of water excites convulsion.* "On suggesting that he should swallow a little water, he seemed to be frightened, and began to cry out. He turned suddenly in bed, and was simultaneously seized with a momentary clonic spasm of the trunk, greatly resembling emprosthotonos; however, by kindly encouraging him, he soon manifested a willingness to accede to my wish, but the sound of the water as it was poured into the teacup, again brought on a similar convulsive action." (Case of Edward Lloyd, aged nearly 11, by Mr. Thornhill, 'Lond. Med. Gaz.,' vol. xvii, p. 270.)—"On our proposing to him to drink, he started up and recovered his breath by a deep convulsive inspiration. . . . . . . On being urged to try, he took a cup of water in one hand, and a spoon in the other. . . . . . With an expression of terror, yet with great resolution, he filled the spoon and proceeded to carry it to his lips; but before it reached his mouth, his courage forsook him, and he was forced to desist. He repeatedly renewed the attempt, but with no more success. *His arm became rigid and unmoveable whenever he tried to raise it* towards his mouth, and he struggled in vain against this spasmodic resistance." (Case of Odell, aged 28, by Dr. Marcet, in 'Med. Chir. Trans.' vol. i, p. 133.)

*The sight of water induces convulsions.* "Sensibility to touch markedly acute; an embrocation to the external fauces produced convulsions; passed urine of a lemon colour, easily, could view it without horror in a black earthen pot; in a glass the sight produced instant convulsions." (Dr. Vaughan's case of Thomas Nourse, aged 14, in Dr. Hamilton's 'Remarks on Hydrophobia,' vol. ii, p. 434.)—"Desirous of cold air, but it constantly renewed his distress; sight of water excited convulsions." (Dr. Vaughan's case of a farmer, aged twenty-five, in ibid. p. 439.)—"Sobbed deeply at the sight of water, turning away with perturbation." (Dr. Vaughan's case of a boy, aged 8 years, bitten by a cat, ibid., p. 441.)—"On water being poured from one basin to another before him. . . . it excited convulsions, and caused him to dash himself against the head of the bed, as if endeavouring to escape from the sight." (Ibid., p. 456. Case of a man, aged 36.)—"Some ale being brought to Dr. Adam while he talked with the patient, he started up from the table at the sight of the mug, and ran away." (Dr. Adams' case of a farmer, aged 40. Ibid. p. 468.)

I shall not now refer to the pathological action of colours, especially of red, on the motor part of the nervous system, because the facts may possibly be disputed. The physiological action is beyond question. The incident excitor influence of odours in inducing convulsions, &c. is, however, well substantiated. So common is this result at Rome, that Sir J. Clark has noticed the fact in his classical work on Climates, and his observation is especially worthy notice, "that it is not disagreeable odours which produce such effects on the nervous system, but the more delicate, and to northern nations, agreable odours of flowers and other perfumes." So that the results cannot be analogous to those induced by stimulants applied to the nostrils.

Having thus adduced facts proving that the sensual nerves are incident excitor, I need only refer to the phenomena of hydrophobia to show farther, that the impressions made on them at their periphery pass on to the central axes, and there induce the necessary changes in the posterior gray matter, so that excito-motory acts shall result.

I have stated that the idea of water, whether obtained through the eye or ear, will excite the hydrophobic gasp and convulsions; it will also excite a conservative act, the patient, when water is presented to him, is horrified, and immediately attempts to remove it. This movement is strictly involuntary, and not the result of sensation; the water is repelled from the lips with a violent spasmodic jerk, and often in spite of the urgent volitional attempts of the patient to the contrary, just as the hand is snatched away from a spark of fire, or the headless frog leaps from the needle. I have already shown that acts strictly involuntary are simply reflex acts, accompanied with sensation, and that consciousness does not invalidate their character. By what channel then can the idea of drinking, originated in the brain by the presence of water, act upon the respiratory muscles, so as to induce gasping, and upon the excito-motor nerves of the head and arm, so as to excite the convulsive removal of the offered cup of water?

The cerebral nerves being analogous to the posterior spinal nerves, and the encephalic ganglia analogous to the spinal ganglia, the spectrum of the cup of water will traverse the optic nerves, and enter the analogue of the posterior gray matter in the brain causing changes, (ideagenous changes,) corresponding to the idea of water; thence the series of excited changes will pass over to the analogue of the anterior gray matter exciting another series, (kinetic changes, *κινητικος*,) by which the necessary groups of muscles are combined in action. If the cerebral ganglia be but a higher development of the spinal, the medullary, and cortical substance must correspond to the white and gray matter of the cord, and if it be acknowleged, (as has indeed been proved beyond question,) that a combined action of sets of muscles, exhibiting a design of conservation may be developed in the spinal cord without the aid of volition, how can we deny the same qualities to the encephalic ganglia, or in other words, to the cerebral hemispheres and their connexions?

We must consider then each half of the encephalon as consisting of two tracts of cortical, and two of medullary substance; the medullary associating ideas and combining muscular movements; the cortical, conducting impressions to the gray matter, giving rise to sensation and perception, and thence to the muscles, exciting motion. That impressions received by the sensitive nerves excite trains of ideas is generally acknowledged, and that the ideas constituting these trains have a connexion with the elementary constitution of the brain is clearly inferrible from the numerous observations recorded, in which the memory has been only partially abolished, as for example in the case recorded by Dr. Abercrombie. In this instance, a lady had lost the recollection of ten or twelve years only; everything previously to that time she remembered quite well, all else she had forgotten. Indeed, since an infinity of muscular acts are already inscribed within the structure of the anterior gray matter of the spinal ganglia, and require only the appropriate sensory impression to rouse them into action, so ideas may be inscribed, and require only sensory impressions to rouse *them.* The posterior gray matter or its analogue in the brain may then be considered as the seat of associations and trains of ideas.

It will be scarcely necessary for me to state in detail, after the preceding remarks, the facts and arguments which may be adduced to prove that the brain, (comprising cerebrum and cerebellum,) is an excitor of reflex acts. Dr. Marshall Hall has relied mainly upon the experiments of Professor Flourens in support of his opinion that the brain is inexcitor, but it will be seen that these experiments consisted simply in irritating the brain by pricking and tearing. Professor Flourens found that if the central axis be irritated mechanically from above downwards, beginning with the hemispherical ganglia or brain, that no spasmodic motions are excited until the tubercula quadrigemina be touched; it is on irritating that point that excito-motory phenomena first appear, and from that point downwards to the cauda equina, they may be produced by mechanical stimulants. Reflex acts do not, however, consist in convulsive movements of the muscles only, nor are they produced most distinctly in the mode adopted by Professor Flourens. Such irritations differ altogether from even the tactile sensations received by the general surface. As every nerve has its proper endowments, and requires the irritant peculiar to itself, to develop the reflex phenomena indicative of design, so the sensory gray matter in which the sensual nerves end must have its proper endowments and peculiar stimuli. Now, no pricking or tearing could induce these changes that depend on the undulations of an elastic medium. The irritant must be much more closely assimilated to the normal excitation. From Dr. Stilling's researches we know that strychnine is an efficient excitant to the gray motor track, and it is more than probable that a skilful application of narcotics to the sensory track in the encephalon of frogs might lead to important results. There are two modes in which the centric excito-motor phenomena of the brain may be studied: first, by considering the action of narcotics circulating with the blood through the brain, as Dr. Hall has considered the phenomena of hydrophobia and asphyxia; and secondly, by analysing the centric phenomena dependent on functional derangements of the encephalon. Examples of both kinds are numerous; of the latter class is the singular case observed by Mr. Wood, and as it is an undoubted example of cerebro-spinal reflex acts, and illustrative of my previous remarks, as to the centric excitation of ideas and combined movements, I shall analyse its principal phenomena. The patient was a young married female nursing an infant aged 14 months. She first had a painful affection of the right side of the face, pains darting from the cheek to the temple and teeth; the incident excitor branches of the fifth were affected. In two or three days, the excito-motor branches going to the orbicularis and levator palpebræ, were implicated, for an involuntary motion of the eyelids then commenced, in which they were opened and shut with excessive rapidity for about fifteen minutes. Then the excito-motor spinal nerves of the right side were implicated, for the movements of the eyelids were instantly succeeded by involuntary motions of the right leg and arm, continuing for about ten minutes. The motions then intermitted for about ten minutes, and recommenced in *all* the extremities with increased violence. But these movements were not mere spasmodic or convulsive jerks; groups of muscles were brought into action. The palms of the hands beat rapidly on the thighs, and the feet on the ground. The fore arms were rubbed incessantly along the thighs, and the radius rotated on the ulna. The arms were at times extended, and the palms turned outwards. Next day the muscles of the trunk were affected, and the patient was suddenly raised from the chair and as quickly reseated. The motions of the eyelids were followed by vomiting, showing that the centric change had extended to the pneumogastric ganglia. The next day the consentaneous action of groups of muscles were still further extended; the centric changes evidently making progress upwards, for in addition to the previous motions she was now jerked from side to side of the couch-chair on which she sat; she had often a sudden propensity to leap upwards, and was impelled into every corner of the room, striking the furniture and doors violently with the hand. Here decided marks of design appear in the movements. On the following day the acts had become rhythmical, and the centric changes had evidently arrived at some portion of the encephalon connected with the *idea of time;* she frequently danced upon one leg, and in the evening the family observed the blows upon the furniture to be more continuous, and to assume the regular time and measure of a musical air. As a strain or series of strokes was concluded she ended with a more violent stroke, marking the time. The next day, the centric change had ascended higher. The rhythmical movements had become more complex, and changed into a graceful dance. But the changes had now reached the *idea of space* as well as of time, for occasionally all the steps were so directed as to place the foot constantly where the stone flags joined to form the floor, particularly when she looked downwards. An analogous result occurred when she looked upwards; she then had an irresistible propensity to spring up and touch little spots on the ceiling. In both these movements, the optic nerve exhibited an incident-excitor function. The tune was now discovered that she danced to; it was the air of the "Protestant boys," popular in the neighbourhood, and she informed Mr. Wood that there was always a tune dwelling upon her mind, which at times becoming more pressing, irresistibly impelled her to commence the involuntary actions. The centric changes here ceased, which had induced this alteration of sensory function, and which had reproduced in fact the idea of the air with such force that it impinged on the motor track, and there excited consentaneous reflex acts, in spite of the utmost volitional effort of the individual. The motions were stopped by interrupting the action of the excitor (the musical air) on the motor track, for so soon as the time was broken, or a continued roll played on drums, the motions ceased. The patient had several relapses; the eyelids and muscles of the face were only affected in some of these, in others, the muscles of the chest, larynx, neck, and back. In one attack she rotated swiftly.

Having traced the progress of the symptoms of this case, I need not recapitulate them, as illustrative of reflex cerebral function. If the brain be indeed the organ of ideas, and the cerebellum of combined movements, the inference is manifest, that they are both excitors of reflex actions. The case is one of many similar recorded by authors: in this, fatigue was felt; in others, the violent efforts did not cause weariness.

What I have just detailed is an example of idiopathic centric change, but encephalic reflex phenomena may be excited by narcotic poisons. An instance of this is related by Mr. Duffin, (Medical Gazette, vol. xv. p. 194,) whose little girl, aged 2½ years, was poisoned by the seeds of stramonium. In about an hour after eating the seeds, symptoms not unlike those of hydrophobia came on. There was " a flushed countenance, wildness of manner, suffused eyes, maniacal expression, ineffectual efforts to vomit, incoherent and rapid utterance, which very soon became wholly unintelligible, screaming, catching at imaginary objects in the air, or rather, striking at them—for it was evident that these spectra were of a frightful nature, since, at the moment of darting out the hand in the direction where the eyes were fixed, she always, suddenly and with great vehemence, withdrew herself, expressed the utmost terror in her look, and then hid her face, at the same time screaming and sobbing violently. Her eye would, to appearance, follow the imaginary object for a moment or two before she made the effort to escape from its supposed approach. She rapidly became furiously delirious, struck at, pinched, or attempted to bite, every person who came near, or any object that was offered to her.

" Within the space of two hours and a half from the time that she must have swallowed the poison, the child had not only lost the power of utterance, but that of voice also. She could now only utter a hoarse croaking sound, alternated with a sonorous, croupy, barking cough; and was unable to swallow, in consequence of the violent spasm which affected the muscles of deglutition when she made the effort. This state of spasm, judging from the nature of the cough, and the croupy character of the inspiration, pervaded the muscles of the larynx. She now knew no person, and had been wholly insensible to surrounding objects for above an hour and half. The pupils were dilated; had been so from the first, and continued in this state till she died. The voluntary power of the extremities was gone, and the limbs were violently agitated by spasmodic twitching and jactitation (not by regular convulsions), alternately with short paroxysms of tetanic spasm (opisthotonos)."

In this, as in the previous example, there is a continued series of reflex phenomena of centric origin, implicating the cerebrum as well as the medulla oblongata, all originating in one cause, namely, the circulation of a poison with the blood, and acting, not mechanically by effusion or pressure, but directly on the intimate organization of the central axis, and developing a succession of changes, commencing with the exaltation and ending with the extinction of its functions. Amongst the insane, especially the idiotic and fatuous, examples of combined excito-motory movements of cerebral origin, are not unfrequent. A male patient in the York County Asylum, aged 44, and fatuous for thirty-seven years, cannot pronounce any word distinctly, nor understand what is said to him. He constantly holds a stone, or other substance, in the palm of one hand, and moves continually, as if slowly waltzing. Mr. Alderson, the resident medical officer, kindly assisted me to time his movements, and we found that he performed twenty steps in fourteen and a half or fifteen seconds, with the greatest regularity, and we measured his steps repeatedly. Another man, aged 34, in a state of dementia, stands for hours together, moving his hands and feet synchronously, in a way not easily to be described. He was found, when timed, to make twenty steps in ten and a half or eleven seconds, with unvarying regularity. In these examples, as in the case of chorea, the cause of the movements was centric; and, as the latter were connected with an idea of time, its seat was undoubtedly cerebral.

There is one other excito-motor phenomenon I would refer to. In hemiplegia, an experiment is occasionally made by nature : a functional change is induced in that part of the central axis devoted to language. In these cases, when the will is directed to the enunciation of one word, as " bread," the individual utters another word, as " boots." Sometimes it is a letter only that is thus mispronounced, as *z* for *p*; and sometimes the words of foreign languages are mixed up in confusion, in spite of the individual's efforts to articulate aright. This phenomenon is analogous to the irregular acts of groups of muscles.

My paper having already extended to so great a length, I will only briefly refer to the instinctive and emotional acts. If the effects of emotions be analysed, it will be found that they act principally upon the excito-motory system, relaxing the sphincters, and inducing vomiting, dyspnœa, sighing, sobbing, gasping, &c. Examples of all these might be adduced. Both the instinctive and emotional acts are essentially conservative; and both so act on the muscular system that a sensation of fatigue is not felt during their action on the motor system. Both may be traced from the simple reflex phenomena to the more compound. Thus, tickling the soles of the feet causes a spasmodic jerking of the limb; but in many instances it will excite violent and involuntary laughter: reflex laughter may also like weeping, sighing, hiccough, gasping, &c., be of centric origin. It is seen in hysterical and hemiplegic cases, and I have myself witnessed it as a sequel of epilepsy from tumour on the cranium; the laughter alternated with weeping, and was accompanied by partial paralysis of the lingual and pharyngeal muscles. In this case, the whole reflex phenomena were of centric origin.

It is only by the theory I have advanced, that we can explain the instinctive acts of animals. Like the purely reflex conservative phenomena, they are altogether dependent on the connate structure of the cerebral ganglia. A young brood of partridges, tended by a bantam hen, will immediately cower and squat motionless, if a stuffed polecat be placed within their view, and they will peck at grain and insects before they have got rid of their shell. Bees will begin to gather wax and construct cells, within twenty-four hours of their being hatched, and before their wings are dry. In all these the acts are in every respect analogous to the compound conservative acts of the true spinal system; the only differences being in the nature of the sensory impression which excites them, in the endowments of the nerves along which the impression is conducted, and in the composition of the central axis.

### Appendix.

I. *On the tone of the muscles.* The state of the muscular system termed tone, is allied in its origin to the muscular contractions of excito-motory movements.

As special impressions on incident excitor nerves give rise to special combined movements, so the general impressions on the whole surface of the body and on the mucous membranes excite the cerebro-spinal ganglia into action generally, and thus a corresponding result is obtained; namely, a general reaction (through the motor nerves) on the whole muscular system. That the cerebral nerves have an important part to perform in the maintenance of this general excitation of the nervous system, and the resulting tone of the muscular system is manifest from the phenomena of sleep. Many of the common muscular acts of the waking state are excito-motory in their nature, as for example, the tension that maintains the lineaments of the face, and keeps the eyelids, head, limbs, and trunk in their usual position. When sleep comes on sensory impressions cease to act on the brain, and the muscles relax. The eyelids then drop over the eyes; the head droops; the limbs become flaccid and uncontracted. If the incident excitor nerves of the abdominal viscera were also liable to sleep, their ordinary functions would be interrupted, and the flexor muscles (so constantly affected in spasmodic affections depending on irritation of the mucous membrane,) would lose that excess of tone they possess over the extensor. If the nerves of the heart and lungs could undergo this change, death would speedily ensue. In the natural condition the incident excitor action of the sensitive branches of the vagus is only diminished in intensity; the heart and chest act more slowly. Sleep appears to be confined to the encephalic ganglia; when it affects the medulla oblongata or spinal ganglia, the change induced is a morbid change. The following may be termed

*A case of sleep of the respiratory ganglia.* A West Indian, a surgeon, consulted Sir Charles Bell. He stated that "on falling asleep, just at the moment when volition and sensibility cease the involuntary motions also stop, with a sensation of death, under which he awakes generally convulsed. His medical friends have sat by him and watched him, and they have found that when sleep is overpowering him, the breathing becomes slower and weaker, the heart and pulse also fall low, and cease to beat as sleep comes on, and after a short time he awakes in terror." (Appendix to Papers on the Nervous System, by Sir Charles Bell. Case 170.) It would appear that incubus or nightmare consists in sleep of the respiratory ganglia.

The tone of the muscular system may be maintained (just as excito-motory acts may be excited) by changes *within* the cerebro-spinal ganglia, or, in other words, by centric changes. We have a remarkable exemplification of this general principle in those examples of somnambulism in which the individual is perfectly insensible to external impressions. In these the nerves sleep; the brain wakes. But the contrary may happen; the cerebro-spinal ganglia may cease to react so as to induce muscular tone, while the incident excitor nerves are awake. Something like this occurs when certain emotions (as fear) excite such violent nervine changes as to interrupt the action and reaction of the central ganglia on the incident excitor and reflex-motor nerves. In such an instance as this, muscular tone is not only destroyed but the contractility of the sphincters is abolished. The action of certain poisons on the central ganglia is precisely analogous. Tartar emetic, tobacco, &c. by their action on the cerebro-spinal axis, destroy the tone of the muscles, more or less completely.

II. *The diffusion of impressions with reference to reflex cerebro-spinal action.* When an impression is made on an afferent nerve an instantaneous change takes place in the gray matter of the ganglion in which the nerve terminates, and this is propagated to the roots of the muscular nerves. But it has been generally forgotten that this is not all; a change passes also along the twigs of the sympathetic nerve connected with the ganglion, and so the secreting as well as muscular and sensory structures have an influence communicated to them. In short, a change is effected in *all* the fibrils entering into the composition of the ganglion. The proofs of this proposition are various: Firstly, it is actually observed to occur in the lower forms of organized matter. Secondly, it has been found by experiment, that the influence of impressions is diffused through a *chain* of connected ganglia, as for example, when the cord of a frog is subjected to experiment. (Vide Stilling's Researches in Br. and For. Med. Rev. vol. XVII, p. 399, and Propositions 12 & 13, p. 400.) Thirdly, pathological observations agree with the results of vivisection. In analysing a case of paraplegia, following a blow on the neck, and detailed by Dr. W. Budd, Dr. Carpenter makes the important deduction "that all influences from impressions on incident nerves are diffused through the cord" (Principles of Human Physiology, 1st ed. p. 132). This principle of the diffusion of influence is applicable as well to the encephalic as to the spinal ganglia. The motor track throughout the cerebro-spinal axis is distinctly influenced by every act of volition, and the whole of that axis, whether sensory, motor, or sympathetic, by every emotion. The action of the heart, for example, is accelerated, as is well known, by very slight muscular efforts; the simple act of rising from the recumbent to the upright posture accelerating the pulse. This diffusion of the volitional influence is seen in diseases of the motor system: in chorea it produces irregular muscular movements; in epilepsy, the motor excitement resulting will prevent the fit.

That influences from emotional impressions are diffused through the whole cerebro-spinal axis, is one of the best-established facts in physiology. The effect of vivid emotions on the functions of the viscera is instantaneous. The skin, intestines, kidneys, liver, heart, salivary and lachrymal glands, and capillaries of the surface, are notoriously influenced by them: Dr. Erdmann, of Dresden, relates a case in his Medical Observations, of a boy whose face, when he was put into a passion, became quite pale on one side and red on the other; and there was an exact boundary along the centre of the face, proving the common union of the sympathetic motor and sensory twigs in the encephalon. The influence of emotions on the hue of the chamelion, and on the colours of certain fishes, strikingly illustrates their operation on the whole system. No class of causes are so influential in exciting convulsions as the emotions, but, like the volitional stimulus, the emotional excitement will *prevent* excito-motory phenomena, and even cure paralysis. Both fear and anger have been known to have this result. It is manifest too, that the diffusion of the influence of emotional impressions is not limited to the true spinal system, or to the ganglia at the base of the brain, for the exaltation or confusion of the understanding, often amounting to insanity and an abolition of consciousness consequent upon their operation, plainly shows that they not only rouse it, but their influence is diffused through the cerebral hemispheres,—the organs of intellect.

Many curious phenomena are singularly illustrative of this diffusion of impressions, and are easily explained by it. Dr. Stilling points out its share in exciting the emotional cries and conservative acts, when disagreeable impressions are made on afferent nerves. (Br. and For. Med. Rev. vol. XVII, p. 139.) The influence of light on the nervous system in maintaining its activity and tone, and preventing sleep is well known. This influence is subject to the law of diffusion. Jungken was acquainted with two persons who were instantaneously seized with asphyxia if light were excluded, or awoke in a state of suffocation if their taper had gone out. A case of this kind is stated in Dr. Forbes's translation of Laennec. In these instances the incident excitor impression of light maintained the activity of the respiratory ganglia, prevented them in fact from going to sleep. The diffused influence of light will produce an opposite effect. Obs. 86, in Bordeu's 'Recherches sur le Pouls,' is that of a very aged female in whom a single ray of the sun or the light of a candle excited an abundant sweat, so that she was obliged to be always in the dark. Many of the phenomena of mesmerism may be explained on the hypothesis of a diffusion of the influence of impression; indeed the theory is as capable of extensive and important applications to therapeutics and hygiene as the excito-motory doctrines.

III. *The substrata of psychical phenomena.* The question necessarily arises how is it that when an impression is thus diffused through the cerebro-spinal axis, certain groups of muscles, the contractions of which constitute instinctive, emotional, consensual, and volitional actions, are excited into energy. The answer must be sought in a knowledge of the histological composition of the cerebro-spinal axis, and of the nature of the bio-molecular changes induced therein, and on the periphery, by the qualities of matter. These adapted acts differ very widely from mere convulsive movements or tetanic spasms, both in their nature and mode of excitement. There is manifestly a mechanism on the periphery from which the sensitive nerves commence, as well as in the centre, appropriate to the inner or ganglionic mechanism. The doctrine of a molecular organization within organized structures, such as that it shall correspond and be appropriate to given stimuli received by appropriate organs, necessarily constitutes the basis of all inquiries into the laws of action in those structures. And there can be no doubt, such is the magnificent uniformity in the immense diversity of creation, that the laws of action of the agent and reagent in vital phenomena, are as definite as those operating on chemical phenomena, could we but effect a sufficiently minute analysis and induction.

It may be useful to state some general principles respecting the *ideagenic* and *kinetic* substrata, alluded to as making up the nervous centres. In the first place, it is to be observed that they are as invariably transmissible from parent to offspring as any other portion of the system, and are subject to the same laws of development; they are therefore as much a part of the animal as its nerves or blood-vessels. This proposition must be steadily remembered as an important clue to an explanation of the origin and mode of action of the substrata in the cerebro-spinal axis. Secondly, these *ideagenic* and *kinetic* substrata may be modified, as any other organ of the body, by intermixture of species or genera; or new substrata may be formed by the reaction of external stimuli on those already existing; or, in other words, new instincts may be acquired and be transmissible. This proposition is scarcely less important than the preceding. Thirdly, these substrata may be persistent as a part of the organism, and continue to be manifested by acts long after the necessity for those acts, as conservative of the individual or race, has ceased. Fourthly, these substrata may be dormant for a lengthened period from the want of a reagent, and appear extinct, but will reappear so soon as the impressions adapted to their action are received by and conveyed along the afferent nerves. Fifthly, as there is a general development of organized beings, as well as of races, those substrata which are common to all will be the most indestructible in each, and the instinctive acts of which they are the basis the most decided and permanent.

The illustration of these propositions need not be numerous. The invariable sameness and permanence of the instincts of the hymenoptera among insects is one of many similar examples. The crossing of breeds of domestic animals and the mixed qualities resulting, is a familiar illustration of the second proposition. Many examples of acquired instincts are on record; several of the best authenticated are detailed by Dr. Carpenter in his 'Principles of General and Comparative Physiology,' 1st ed. § 549. The following is an interesting fact of this kind. A troop of cavalry, which had served on the continent, was disbanded in York. Sir Robert Clayton turned out the old horses on Knavesmire to have their run for life. One day, while grazing promiscuously and apart from each other, a storm gathered, and when the thunder pealed and the lightning flashed, they were seen to get together, and form in line in almost as perfect order as if they had had their old masters on their backs. Fishes can acquire these substrata. Mr. Ellis, in his 'Polynesian Researches,' says, that he has frequently seen a large eel come to the surface of the water when his master (a young chief) *whistled,* and take food from his hand. The persistence of these substrata is shown by the instinctive actions of the dog when about to go to rest. The best bred Blenheim spaniel will scratch his cushion and turn himself round and round (the instinctive act of the wild dog) before going to rest. Like the fox, domestic dogs will hide their food in the earth. A friend of mine lost two fowls, and it was only after some time, on finding the legs sticking out of the ground, he discovered that a handsome Blenheim bitch in his possession had killed and hid them. The domesticated squirrel will hide his nuts in the hay of his cage, but he will also place them on the carpet, or a mahogany table, and giving them a few pats, (just as when hiding them in the hay,) leave them. The reexcitement of dormant substrata is illustrated by the instincts of the wild horses in South America. The following observations, made by Sir R. H. Bonnycastle, in his work on Canada, strikingly exhibits the existence of substrata dormant in man, until the appropriate stimulus is received: "The best specimen of an Indian Missionary I am acquainted with in Upper Canada forgot all his instruction, all his acquired feelings and habits, when he witnessed with me the war-dance of heathen and perfectly savage warriors. He had been carefully educated from a boy, was modest, intelligent, and well-bred.... ....Yet he grinned with savage delight at this exhibition of untutored nature."

The fifth proposition regarding these substrata is one of most extensive application. Just as in man certain organs are rudimentary, so also certain of these substrata are rudimentary; just as the osteology of man is formed on one general type, varied only to suit his mode of existence, so also these substrata are based on a fundamental type varied in like manner. And just as monstrosities and physiological changes occur, marking a retrograde step to a lower form of organization, so are the substrata of lower instincts developed and excited into action. A remarkable instance of this has been recently published. "A perfectly idiotic girl, in Paris, having been seduced by some miscreant, was delivered of a child without assistance. It was found that she had *gnawed* the umbilical cord in two; in the same manner as is practised by the lower animals. It is scarcely to be supposed that she had any idea of the *object* of this separation." (Dr. Carpenter's Physiology, 1st ed. p. 219.) Thus the kinetic and ideagenous or sensorial textures of the ganglia of all animals are interwoven with those of the human organization. It is only by a hypothesis of this kind that we can explain various instinctive acts in man. The incident excitor action of water on the respiratory organs is an anomaly, unless we can attribute it to a substratum belonging to a lower grade of development.

The qualities of water are not stimulating to the skin; its contact excites no pain or irritation on the general surface, and yet, when dropped on the head or chest, as in a shower-bath, the larynx is immediately closed, and an instinctive feeling of terror excited. When the substratum corresponding to the impression it makes on the afferent nerves of the head, body, and thorax is morbidly excited, as in hydrophobia, or certain forms of spasmodic asthma, the gentlest contact of a blander matter than water, but inducing a similar impression—the air we breathe —will excite the horrid feeling of impending death from suffocation, and instinctive terror in its wildest form.

IV. *Probability of the theory of substrata appropriate to psychical phenomena.* This theory of a nidus or substratum, for the reception of impressions and the excitation of ideas and acts, is by no means new. Prochaska adopts it distinctly with reference to the spinal cord; and Hooke, Locke, Haller, and others, with reference to the brain. Haller says expressly, "Eas mutationes in sensorio conservatas *ideas* multi, nos vestigia rerum vocabimus, quæ non in mente sed in ipso corpore, et in medulla quidem cerebri ineffabili modo incredibiliter *minutis notis* et copiainfinita inscriptæ sunt." Hooke went even so far as to theorize on their formation, and estimate the numbers that could be made in a day. The theory flows necessarily from the proposition that the brain is the organ of the mind; it is also a necessary inference from all that we know of the functions of the nervous system. The principal objections have been, first, that it leads to materialism, and secondly, that the immense multitude of ideas and consensual acts renders such a texture of the constituent fibrils impossible. I shall defer a notice of the first objection, which can easily be shown to be quite groundless. This second arises in the mind, because we have neither sufficiently examined nor contemplated the more recondite properties of matter. We know that the divisibility of matter is so great as to elude all our means of research, and to give rise to the idea of its *infinite* divisibility. The microscopic forms of organized matter are wondrously minute; and when we know certainly that beings invisible to the naked eye have structures as diverse as those of the largest animals, and as perfectly adapted to their modes of existence, the histological constitution of which defies even the powers of imagination, there can be no ground for surprise at the infinite variety of ideas interwoven into the connate structure of the cerebro-spinal axis, or written during life on the brain. The sensible points of the retina, according to Weber and Smith, measure no more than the 1-8000th inch in diameter. If, adopting the views of Mr. Solly, we consider the convolutions of the brain as made up of an extensive surface of cineritious neurine, we may estimate the number of ideas, the substrata of which may be contained in a square inch, as not less certainly than 8000; and as there must be an immense number of square inches of surface in the gray matter extended through the cerebro-spinal axis of man, there is space sufficient for millions.

V. *The consensual movements.* The harmonious and consentaneous action of muscles and groups of muscles (just as the purely reflex and instinctive acts) differs from mere spasmodic contractions. The evolution of consensual acts from the lower to the higher forms of development, takes place also after the same laws. In the primary forms, the irritability of the muscular fibres excited them according to a fixed principle of consentaneity and adaptation. The hollow muscular tubes, the heart, arteries, and urinary bladder, are instances of the lower forms in which groups of *fibre* act consentaneously. Next come consentaneous action of groups of antagonizing *muscles*, flexors and extensors, pronators and supinators, adductors and abductors. The spasms of tetanus and epilepsy result from a morbid influence on the substrata of these consentaneous acts. To a higher grade of this kind belong the substrata of the class of co-ordinate muscular acts observed in rotation, progression, retrocession, flying, swimming, and the like, the general movements of the lower vertebrata. Allied to these are the substrata which determine the gait, bearing, language, tone of voice, expression, &c. of the individual, and which bring them into relation with the emotional and instinctive acts. They differ from the preceding in this, that they are due to special groupings peculiar to the individual or the race. They follow, however, the law of transmission from parent to offspring, guiding the other substrata referred to above. A peculiar gait, a certain kind of frown, a hitch of the shoulder, a tone of voice, are all the result of co-ordinate muscular acts taking place independently of the volition of the individual, and almost always without his consciousness, and appear as certainly in the offspring as any other corporeal peculiarities. Co-ordinate or consensual substrata, like those ministering to the instincts, may also be acquired and appear as *habits;* and these may also be transmitted, though not usually. Lastly, the substrata of the highest co-ordinate movements, namely, those dependent on the intellect, and seated in the cerebral hemispheres, are the substrata on which the acts of speaking, singing, writing, painting, music, &c., and the practice of the manual employments, depend. These are almost always acquired, and seldom transmitted; but on this point, especially with reference to the last class, observations are wanting. According to these views, any attempt to localize the substrata of the coordinate or consensual acts would be futile. Like those of the instinctive and emotional movements, they extend through the whole cerebro-spinal axis. The stimuli that excite them are of course local in their origin, and as diverse in their character as their origin.

VI. *The association of ideas.* Like the association of movements, the true explanation of the association of ideas is to be found in the doctrine of the reflex functions of the brain. The mode of action of the sensory gray matter is strictly analogous to that of the motor gray matter, both with reference to its substrata and the diffusion of afferent impulses through it. Insanity and dreaming present the best field for investigating the laws of that extension of action from one portion of the brain to the other, by which ideas follow each other in sequence. An interesting example for study is now in the Retreat near York. This person seems utterly will-less. He expresses the ideas as they spontaneously arise in associated sequence, the combinations being singularly varied, but traceable to a common root, or centre of impulse. Researches of this kind, whether instituted on the insane, the somnambulist, the dreamer, or the delirious, must be considered like researches in analytical chemistry. The reagent is the impression made on the brain; the molecular changes following the application of the reagent are made known to us as ideas. In chemical analysis we know the molecular changes only by the change in form, refractive powers, and other circumstances, induced by the reagent; in cerebral analysis we *feel* the change, or observe its results on the efferent nerves. It is very probable that only on researches of this kind can a scientific spiritualism be established, and through them the link seized that connects the spiritual with the material world.

VII. *The psychical position of man in creation.* The law of unity of type and function in animals, applied in the preceding pages to the function of the cerebro-spinal axis in man, has shown (what is necessarily deduced from the law itself) that the transition of structure and function is gradual, and consequently, no strong line of demarcation can be drawn between the manifestations of its various functions. The automatic acts pass insensibly into the reflex, the reflex into the instinctive, the instinctive are *quasi* emotional, the emotional are intellectual. This gradation of structure and function observed in the nervous system, is observed also with reference to all other structures of his body. Man is at the head of a vast ascending scale of animal life, so extended in its connexions downwards, that for the present purpose it may be considered as infinitely extended. With our existing knowledge of the uniformity of the laws of creation, the deduction is absolutely incontrovertible, that the scale of being is not truncated at man, and that beyond him there cannot be a dark, unpeopled void. The law of gradation of development vigorously pushed to its legitimate conclusions points out an infinite gradation of being *above* and *superior* to man. That we cannot see such beings, nor demonstrate their existence is a necessary, result of our position in the scale, and no proof whatever of their non-existence. The worm knows nothing of man, his works, or his actions: nothing of the sun or the stars, or of the beings swarming around it: and so with reference to the spiritual world, the world around and above *us*—our organs may be, and doubtless are, as imperfect as those of the worm with reference to the world around and above *it.* Man is then at the foot of another scale of beings, the highest of which, *at least,* as far transcends man, as man transcends the zoophyte. This proposition, I repeat, is the unavoidable inference from our present physiological knowledge, and is a complete answer to those good, zealous, but not wise men, who think science leads to scepticism and irreligion. It leads to a rational faith utterly opposed to arrogant infidelity.

York; *December* 24, 1844.

